# Aboriginal and Torres Strait Islander family access to continuity of health care services in the first 1000 days of life: a systematic review of the literature

**DOI:** 10.1186/s12913-020-05673-w

**Published:** 2020-09-03

**Authors:** Nina Sivertsen, Olga Anikeeva, Janiene Deverix, Julian Grant

**Affiliations:** 1 College of Nursing and Health Sciences, Flinders University, GPO BOX 2100, Adelaide, South Australia 5001 Australia; 2 School of Public Health, Adelaide Health and Medical Sciences Building, The University of Adelaide, North Tce, Adelaide, SA 5005 Australia; 3 Manager Aboriginal Services, Child and Family Health Service, Women’s Children’s Health Network, 295 South Terrace, Adelaide, SA 5000 Australia; 4 School of Nursing, Midwifery & Indigenous Health, Charles Sturt University, Panorama Ave, Bathurst, NSW 2795 Australia

**Keywords:** Continuity of care, Aboriginal, Maternal-child health, Prenatal care, Antenatal care, Infant

## Abstract

**Background:**

Aboriginal women and their infants experience significant disadvantage in health outcomes compared to their non-Aboriginal counterparts. Access to timely, effective and appropriate maternal and child healthcare can contribute to reducing these existing health disparities. However, accessing mainstream healthcare services often results in high levels of fear and anxiety, and low attendance at subsequent appointments among Aboriginal women, due to inefficient communication, poor service coordination and a lack of continuity of care.

**Methods:**

This integrative literature review sought to explore factors that contribute to continuity of care and consider service features that contribute to positive care experiences and satisfaction with care received by Aboriginal women and their infants.

In total, 28 studies were included in the review and were thematically analysed using Braun and Clarke’s six steps of thematic analysis. This was followed by a collaborative, computer-assisted qualitative analysis, which resulted in the emergence of five key themes: lack of continuity of care, impact of lack of continuity of care, continuity of care interventions, impact of continuity of care interventions, and strategies to improve continuity of care.

**Results:**

Most studies focused on health services in rural or remote Aboriginal communities and there was a lack of documented evidence of continuity of care (or lack thereof) for Aboriginal women living and birthing in regional and metropolitan areas. The majority of studies focused explicitly on continuity of care during the antenatal, birthing and immediate postnatal period, with only two studies considering continuity through to an infant’s first 1000 days.

**Conclusion:**

The review highlights a lack of studies exploring continuity of care for Aboriginal families from the antenatal period through to an infants’ first 1000 days of life. Included studies identified a lack of continuity in the antenatal, peri- and postnatal periods in both regional and metropolitan settings. This, along with identified strategies for enhancing continuity, have implications for communities, and healthcare services to provide appropriate and culturally safe care. It also marks an urgent need to incorporate and extend continuity of care and carer through to the first 1000 days for successful maternal and infant health outcomes for Aboriginal peoples.

## Background

Compared to non-Indigenous Australians, Aboriginal Australians experience a significant level of disadvantage in health, life expectancy, education, employment and living standards [1–8]. These disparities are evident when considering maternal and infant health outcomes, with higher rates of gestational diabetes and smoking during pregnancy among Aboriginal women, rates of preterm birth and low birth weight nearly double among Aboriginal babies, perinatal mortality rates of Aboriginal infants 50% higher than those of non-Aboriginal infants, and maternal mortality rates of Aboriginal women nearly three times higher compared to their non-Aboriginal counterparts [1, 5–21]. Maternal and infant health outcomes worsen with increasing remoteness, due to challenges in health service provision and delivery [6]. This disproportionately affects Aboriginal women and their infants, as 26% of Aboriginal births occur in areas classified as remote or very remote, compared to only 2% of non-Aboriginal births [1, 10, 12, 13]. The factors contributing to the observed disparities are complex and include poor access to culturally appropriate health services, sustained institutional racism, lower educational attainment, poverty, and the continuing effects of colonisation [7, 10, 13, 21, 22].

Healthcare services can contribute to the reduction in existing health disparities between Aboriginal and non-Aboriginal women and their infants, through the provision of timely, effective and appropriate maternal and child healthcare [9, 11, 17, 19, 23, 24]. Health services delivered by a suitably trained and qualified professional throughout pregnancy, birth and the postpartum period can reduce maternal and infant morbidity and mortality, particularly among women whose health status is poor [25].

Among Australian Aboriginal women, poor uptake of health services is associated with geographic isolation, cost, language barriers, lack of trust, previous negative experiences and culturally inappropriate or unsafe delivery and practices [9, 20, 26, 27]. Aboriginal women are less likely than their non-Aboriginal counterparts to attend mainstream health services, commence antenatal care at the recommended time, and attend the recommended number of antenatal visits [10, 19, 28]. This is in part due to maternal and infant healthcare in remote communities being logistically complex and fragmented, involving multiple transfers of care among multiple providers and sectors of the health system, resulting in a lack of continuity of care, poor service coordination and inefficient communication between service providers [5, 29]. Furthermore, Aboriginal women frequently perceived available services as culturally unsafe. A biomedical model of care underpins most mainstream health services in Australia, which can be at odds with traditional Aboriginal ways of giving birth [5, 11, 20, 22, 30]. Healthcare professionals are often inadequately trained and underprepared to work cross-culturally, further compounding the situation [30]. Consequently, accessing maternal and infant healthcare services often results in high levels of fear and anxiety, and low attendance at subsequent appointments among Aboriginal women [11, 27].

Continuity of care is a phrase identified primarily in midwifery practice that refers to service models that incorporate continuity of services and/or continuity of carer across antenatal, labour, birthing and post-natal care. (see for example 43). Continuity of care is reportedly experienced as more culturally safe than siloed care and can result in greater uptake in health care during the perinatal period [31, 32]. Where health disparities continue to exist for Aboriginal women and infants it is essential to explore the factors that contribute to extensions of this continuity to include the first 1000 days of life. This literature review explored factors that contribute to continuity of care as Aboriginal families transition from antenatal care through to child and family health care across the first 1000 days of life. Further, it explored service features that contribute to positive care experiences and satisfaction with care.

## Methods

### Search strategy

The purpose of this integrative literature review was to review, evaluate and synthesise what is known around continuity of care for Aboriginal families in child and family health services in Australia. An integrative literature review systematically searches, critiques and summarises relevant literature [33]. The review was performed according to the PRISMA Statement and checklist and a four-phase flow diagram (Additional File 2). The aim of the PRISMA Statement is to help authors improve the reporting of systematic reviews and meta-analyses [34]. Searches were performed on Scopus and PubMed and were limited to articles published from 2008 onwards. The search terms included in the search are detailed below:

#### Scopus search

“reproductive health services” OR “maternal-child health” OR “prenatal care” OR “antenatal care” OR “postnatal care” OR infant OR “continuity of care” OR “Indigenous health service” AND Aboriginal.

#### PubMed search

((“reproductive health services”[MeSH Terms] OR reproductive health services [Text Word]) OR (“Maternal-Child Health Centers”[MeSH Terms] OR maternal-child health centers [Text Word]) OR (“Prenatal Care”[MeSH Terms] OR antenatal care [Text Word]) OR (“Postnatal Care”[MeSH Terms] OR postnatal care [Text Word]) OR (“Infant”[MeSH Terms] OR infant [Text Word]) OR (“Continuity of Patient Care”[MeSH Terms] OR continuity of care [Text Word]) OR (“Health Services, Indigenous”[MeSH Terms] OR Indigenous health service [Text Word])) AND (“Oceanic Ancestry Group”[MeSH Terms] OR Aboriginal [Text Word] OR Torres Strait Islander [Text Word])

Additional articles were identified through initial background searches on Google Scholar, as well as hand-searching the reference lists of included articles. These searches yielded a total of 2935 results.

### Excluded studies

Identified articles were excluded if they were duplicates (*n* = 1147) or did not focus on maternal and child health services used by Aboriginal people in Australia based on an initial scan of each article’s title and abstract (*n* = 1691). The remaining 97 full-text articles were examined to assess their suitability for inclusion in the review. Articles were excluded if they were study protocols, policy perspectives and position papers, discussions of the risk factors for specific diseases or practices, descriptions or evaluations of specific programs or interventions, or reports of pregnancy or birth outcome trends and statistics (*n* = 30). The remaining 67 articles were individually assessed by the authors to determine if continuity of care was explored at any point across the first 1000 days. Following discussion the authors agreed on exclusion of a further 39 articles that did not discuss continuity of care or continuity of care provision at any period across the first 1000 days. In total, 28 articles were included in the review (see Additional File 1 ‘Articles included’ and Additional File 2 ‘Literature Flow Diagram’).

### Included studies

Included studies employed a range of methods, with the majority using mixed methods incorporating qualitative analysis of interview data and quantitative analysis of clinical data. Also included were qualitative studies with interview, focus group and questionnaire data. A small number of included studies were quantitative, including cohort studies and cross-sectional audits of existing data. The remaining studies were reviews of the literature and descriptions of programs or interventions that included background performance and outcome data.

### Review process

Articles and data were initially analysed by authors NS, OA and JG separately, using the six steps of thematic analysis by Braun and Clarke [35] to identify broad concepts and themes. This individual analysis was followed by computer-assisted qualitative data analysis using *NVivo 12* to enable team-based coding and collaboration [36]. Jackson and Bazeley’s framework for NVivo-guided thematic analysis was applied to re-analyse the article data as outlined in (Table 1) below [37].

**Table 1:**
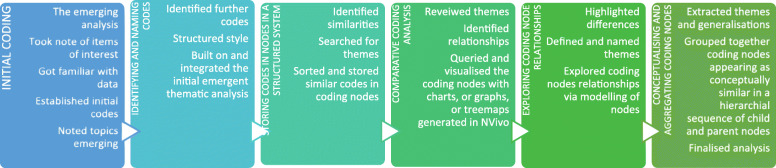
Steps of NVivo guided thematic analysis inspired by Jackson and Bazeley [37]

The collaborative data analysis processes resulted in five key themes; lack of continuity of care, impact of lack of continuity of care, continuity of care interventions, impact of continuity of care interventions, and strategies to improve continuity of care.

## Results

### Study settings

Of the studies included in the review, most (*n* = 20 of total 28 studies included) were conducted in remote Aboriginal communities in the Top End of the Northern Territory or across both metropolitan and rural and remote settings in Australia [1, 5, 6, 8, 10, 13, 14, 19, 20, 24, 26, 27, 29, 30, 38–43]. The studies conducted in the Top End of Australia predominantly focused on Aboriginal community-controlled health services and remote health centres [1, 5, 6, 10, 19, 29, 40], and, in some instances, also included a regional hospital where Aboriginal women relocated for birth [5]. The studies that were conducted across multiple locations usually included a metropolitan or regional hospital as well as a number of remote health centres and explored whether there were links or established referral pathways between these services [1, 5, 6, 8, 13, 19, 24, 29, 44].

The remaining studies were conducted exclusively in metropolitan settings (Sydney, Brisbane, Perth and Adelaide) [11, 26, 39, 44–46] or rural towns in New South Wales and Queensland [14, 27, 38]. These studies were predominantly focused on specific Aboriginal birthing or Midwifery Group Practice programs within hospitals, which provided antenatal care to Aboriginal women within a culturally safe environment and were typically staffed by female obstetricians, Aboriginal midwives and Aboriginal Liaison Officers. A further five studies were review articles examining continuity of maternal health services among Aboriginal women and their infants in Australia [4, 7, 12, 19, 30]. One explicitly included maternal and well child health services [4].

### Key themes

#### Lack of continuity of care across the first 1000 days

Studies included in this review reported a general lack of continuity of care and continuity of carer within maternal and infant health services available to Aboriginal mothers and their babies [1, 4–6, 13, 19, 29, 30, 42, 43, 45, 47]. Lack of continuity of care and carer were especially common in healthcare services without dedicated Aboriginal antenatal and birthing programs and interventions [1, 4, 6, 10, 13, 27, 29, 30, 42, 45], and were more commonly observed within hospitals than remote health centres [10, 45].

Aboriginal women were particularly disappointed with a lack of continuity of care during labour and birth, as well as postnatally [44]. Similarly, continuity of care was compromised for Aboriginal women who presented to health centres with non-pregnancy related concerns during their pregnancies, as they were treated by a different clinician [10]. This lack of continuity of carer, in some cases after experiencing continuity of carer antenatally, contributed to Aboriginal women feeling abandoned and uncared for [44].

Conversely, one study reported that some Aboriginal women who had been offered a continuity of care model of care delivered by an Aboriginal Maternal Infant Care (AMIC) worker declined the service, stating that they did not wish to be treated by a known health care provider [45]. The decision to opt out of a continuity of care model was primarily motivated by privacy and confidentiality concerns [45].

Midwives highlighted a lack of continuity of care within hospitals, especially in large organisations where midwives were particularly time poor and hospital policies and procedures were inflexible [45]. Health care providers expressed concerns about the lack of continuity of care in early childhood services, particularly with regard to access to a Maternal and Child Health nurse to support a coordinated, culturally responsive approach to service delivery [42].

The observed lack of continuity of care and carer was attributed to inappropriate or inadequate resourcing of remote health services, poor care coordination, poor discharge documentation and communication between hospitals and remote health centres, lack of Aboriginal leadership, a focus on a western model of care provision, attitudes and practices of clinicians, time constraints placed on midwives and other health care providers, staff turnover and rotation, and inflexible hospital policies and procedures [1, 6, 8, 10, 44, 45].

#### Impact of lack of continuity of care

Lack of continuity of care and carer impacted Aboriginal women’s experiences of and satisfaction with the care they received and influenced their and their infants’ health outcomes. Lack of care continuity was viewed by midwives as a key barrier to effective care provision within the mainstream health system [45]. A lack of continuity of care has been shown to affect communication and quality of care in antenatal and postnatal services for Aboriginal women [4]. This can in turn influence health outcomes, with fragmented care being shown to increase medical risks and compromise patient safety, leading to adverse outcomes for Aboriginal women and their infants [4].

#### Continuity of care interventions

A number of the included studies described interventions or programs that have been implemented in hospitals and other health care settings to improve continuity of care and carer for Aboriginal mothers and their infants. These programs typically focused on improving continuity of care through ongoing contact throughout pregnancy and birth with a primary midwife, an Aboriginal midwifery student, a district medical officer or AMIC worker [5, 8, 10, 11, 14, 20, 24, 26, 38–41, 44, 46]. Features of these programs that were most highly valued by Aboriginal women were having a single known care provider throughout their pregnancy, strong community links, and being controlled by Aboriginal communities [5, 12, 14, 39].

Continuity of care following birth was not discussed in detail and did not feature as a component of most continuity of care programs. A small number of studies specifically focused on programs that sought to improve continuity of care postnatally [38, 39]. For example, the Malabar Community Link Service in metropolitan Sydney provided continuity of care for Aboriginal women and their infants postnatally, by referral to child health services following discharge after birth and access to a known care provider who Aboriginal women could call with their queries [39].

#### Impact of continuity of care interventions

Continuity of care and continuity of carer were highly valued by Aboriginal women. Having both face-to-face and telephone access to a single care provider who was well known to the woman and who “knew their story” and could act as their advocate was very important to Aboriginal women [5, 14, 39, 44, 47, 48]. In particular, Aboriginal women valued care provided by another Aboriginal woman, such as an Aboriginal midwifery student or AMIC worker, which had a positive impact on cultural appropriateness [8, 14].

Programs that offered continuity of care through antenatal and birthing services resulted in greater acceptability of care among Aboriginal women and greater satisfaction with the quality of maternity care they received [5, 27, 43, 44, 49]. Continuity of care programs appeared to have a positive impact on maternal and infant health outcomes, including improvements in antenatal attendance, better monitoring and management of risk factors, lower rates of preterm birth, higher infant birth weight, and lower perinatal morbidity and mortality [19, 24, 26, 41, 44, 47–49]. However, the methodological quality of studies reporting improvements in maternal and infant health outcomes has previously been assessed as weak and therefore these findings should be interpreted with caution [4].

#### Strategies to improve continuity of care

Included studies put forward numerous strategies to improve continuity of care for Aboriginal women and their infants, including in mainstream healthcare settings that do not have dedicated Aboriginal maternal and child health programs. The importance of establishing and maintaining designated leadership positions, such as discharge coordinators, was viewed as a means to improve communication and handover processes between hospitals and remote health services [29]. In order to expand the role of Aboriginal health care providers in mainstream health services, partnerships should be established with universities and Aboriginal communities to improve education and encourage employment of Aboriginal staff in caseload midwifery models of care [14]. Health services should focus on improving communication and building stronger and more trusting working relationships between midwives, Aboriginal health workers and Aboriginal families [45, 47–49]. This can be supported through ongoing cultural competency training for staff and greater flexibility in the application of hospital rules and regulations to support culturally safe care provision [11].

Aboriginal women should be actively engaged in the design and delivery of maternity care, and programs designed to improve continuity of care should work with Aboriginal women on identified needs to strengthen outcomes [46]. Efforts should be made to ensure that appropriate maternity care is available as close to Aboriginal women’s homes as possible. Where this is not practicable, Aboriginal women from regional and remote areas should have access to AMIC workers to improve continuity of carer, particularly in instance where women relocate to a large metropolitan hospital for birth [11].

## Discussion

Most of the studies included in this review focused on health centres in rural or remote Aboriginal communities or programs targeting continuity of midwifery care for Aboriginal women between their remote or regional homes and metropolitan and regional hospitals. Overall, this review illuminates that there is a lack of documented evidence of continuity of care (or lack thereof) for women birthing in both regional and metropolitan areas in which they also live. Of note, the majority of studies focussed explicitly on continuity of care during the antenatal, birthing and immediate postnatal period. There were only two studies incorporating research regarding experiences of continuity during an infant’s first 1000 days [38, 39]. There was an absence of research identifying service usage and transitions throughout this period. This includes access and engagement with culturally focussed and/or mainstream health, education and welfare services. This review also found that the inclusion of the first 1000 days are imperative to demonstrate that successful family care must have continuity through pregnancy, before, during and after birth up until child is well into toddlerhood, and cannot be isolated into separate stages of antenatal, peri- or postnatal care.

The first 1000 days movement was established to facilitate change to improve global under-nutrition [50]. Drawing on evidence for nutrition and support of neonates to women of reproductive age [51], the movement promotes development across four domains. These include nutrition for brain development, better health, and equity for reaching potential and economic prosperity [50]. While nutrition is vitally important, Aboriginal and Torres Strait Islander leaders in Australia have identified that broader, holistic and cultural perspectives of health and wellbeing are required to facilitate change for Aboriginal children [52]. This is more important now than ever given that the infant mortality rate for Aboriginal children in 2018 was double that of non-Aboriginal children, and the gap between the two rates has widened [53].

Studies included in this review focused primarily on maternity services in remote areas and their links to regional areas. Approximately 80% of Aboriginal birthing mothers do not live in remote or very remote areas [54]. This means that they are living in and accessing health services in regional areas (46%) or major cities (34%) [54]. Very little is known about how mainstream birthing units and Aboriginal Community Controlled Health Organisations (ACCHOs) are connected during the birthing period in these areas. Even less is known about how these are linked with well child health services, either ACCHOs or mainstream services supporting critical growth and development until an infant’s second birthday.

The Nurturing Care Framework (NCF) was launched in 2018 through collaboration between the World Health Organisation (WHO), the United Nations Children’s Fund (UNICEF), the World Bank group, the Partnership between Maternal, Newborn and Child Health and the Early Childhood Development (ECD) action network [55]. It identified pregnancy to age three as a critical period for growth and development. The life course is shaped by early childhood experiences that directly shape the structure and functioning of the infant’s brain [56]. These experiences include the nature of care and enrichment. The research is clear that negative experiences including poor maternal attribution, abuse and environmental insult cause maladaptive structural changes that persist into adulthood [57]. Health outcomes in these early years have long been known to shape adult health and wellbeing, educational outcomes, employment and life expectancy [58].

Parental capacity to provide nurturing care is strongly linked to the social determinants of health and experiences of health inequity and poverty [59, 60]. Recent Australian data indicate that 46% of Aboriginal birthing mothers live in the lowest socioeconomic areas, 52% are aged below 24 years and are 8 times as likely as non-Aboriginal mothers to be teenagers [54]. To enact the NCF and 1000 Days Australia [61, 62] these families require targeted interventions that are experienced as culturally safe and continue after the neonatal period. This review identified that continuity of care in the provision of maternity services appeared to have a positive impact on maternal and infant health outcomes, including improvements in antenatal attendance, better monitoring and management of risk factors, lower rates of preterm birth, higher infant birth weight, and lower perinatal morbidity and mortality, and was the preferred approach for Aboriginal families. To meet the recommendations of the NCF the same lens on continuity needs to be applied to ongoing care through to the early years. It is known that disadvantage is cumulative. Further research is needed to identify how to disrupt this accumulation across the critical early years [54], specifically the part that continuity of care and carer must play in this period of the first 1000 days of beginning life.

This study identified that continuity was hampered by structural issues such as inadequate resourcing, inflexible policies and procedures, time constraints, provision of care from a western biomedical position, and individual factors such as poor communication and unhelpful attitudes and practices of clinicians. Even though much of the research was conducted with Aboriginal and community-controlled health services, these services remain situated within national and jurisdictional biomedical systems of care. These systems are fragmented, with services across the first 1000 days being siloed and subject to disciplinary scopes of practice. Western biomedical models of care provision do not support or maintain an approach to equity in care [63]. Aboriginal perspectives are often relegated to the margins of health care systems and policies, and there is a call for ‘two-eyed seeing’ [64], which would mean that Aboriginal and Western world views of health, illness and child wellbeing, including health care systems, need to come together and collaborate. A two-eyed way of seeing means that one learns to see from one eye the strengths of Aboriginal knowledges, and from the other eye, the strengths of Western knowledges, but most importantly to use both eyes together for the benefit of all. Two-eyed seeing honours multiple perspectives and realities as a gift acknowledged by many Aboriginal peoples [65, 66]. Two-eyed seeing can be applied in the Australian maternal and well child health services, for example by implementing health navigators contributing to continuity of care for Aboriginal families journeying the beginning life.

The NCF calls for systemic and policy change for health care equity with targeted approaches for infants and children most in need. Actions for change are clearly articulated in both the National Framework for Health Services for Aboriginal and Torres Strait Islander Children and Families and the National Safety and Quality Health Service Standards (NSQHSS) User Guide for Aboriginal and Torres Strait Islander Health [67, 68]. Key elements of service delivery include continuity of care and a place-based model for collaboration and integration of services [67]. Further, it identifies multidisciplinary collaborative team-based approaches, well-resourced, highly skilled and competent workforce, holistic care and flexible service delivery [67]. A call to strengthen links between primary care and the acute sector to enable continuity of care is reiterated in the NSQHSS User Guide for Aboriginal and Torres Strait Islander Health in addition to explicit directions to work in partnership (Action 2.13), address the specific health needs of Aboriginal and Torres Strait Islander peoples (Action 1.2), implement and monitor targeted strategies including the allocation of resources (Action 1.4), and improving cultural competency at system structure and individual levels (Action 1.21) [68]. If these directions have existed since 2016 the results of this review indicate an urgent need for research into continuity of care and approaches to care that support optimal growth and development in the early years.

Policymakers must step up; here is an opportunity to invest in future health care. Says Professor Kerry Arabena: ‘The First 1000 Days between a woman’s pregnancy and her child’s second birthday offers a unique window of opportunity to shape healthier and more prosperous futures’. [69] Arabena talks about a First Nations model aimed at strengthening all families so they can give their children the best start in life [52, 61]. Although Elders, community members and early childhood program developers work to provide coordinated, comprehensive, culturally informed interventions to support families [62], looking at health care holistically we need to examine the health care systems and workforce structure – at present even though we know best practice from research, health care systems, structure and workforce are not providing optimum health care outcomes without a focus on continuity of care beyond the perinatal period.

### Limitations

This review was limited by the available published literature and therefore does not reflect the scope of the problem that lack of continuity of care presents. Due to the sparse data and the lack of data from mainstream health services, the outcomes of the retrieved studies may not be generalisable to the entire Aboriginal and Torres Strait Islander populations in Australia. The authors are aware of much work being undertaken at community and service level for practice improvement that is not represented in formal research literature.

## Conclusions

This literature review provides a picture of continuity of care, or lack thereof, for Aboriginal families with infants accessing care through pregnancy and after birth during the window to life from conception to age two within health services in Australia. The main themes of lack of continuity of care, the impacts of this and strategies for achieving continuity of care all have implications for communities, members of communities and the ability of healthcare services to provide appropriate and culturally safe care. This review contributes to inform decision makers around best practice and models of care for Aboriginal families and their babies. This literature review highlights the importance of incorporating continuity of care for successful outcomes. This is an important focus area for future research.

## Supplementary information

**Additional file 1.** 28 Articles CoC Literature Review

**Additional file 2.** Prisma Literature Flow Diagram

## Data Availability

The datasets used and/or analysed during the current study are available from the corresponding author on reasonable request.

## Abbreviations

ACCHOs: Aboriginal Community Controlled Health Organisations
AMIC: Aboriginal Maternal Infant Care
ECD: Early Childhood Development (ECD) action network
NACCHO: National Aboriginal Community Controlled Health Organisation
NCF: Nurturing Care Framework
NSQHSS: National Safety and Quality Health Service Standards
UNICEF: United Nations Children’s Fund
WHO: World Health Organization
