# Supplementary material for: Aboriginal and Torres Strait Islander family access to continuity of health care services in the first 1000 days of life: a systematic review of the literature
Source: BMC Health Serv Res. 2020 Sep 3;20:829. doi: 10.1186/s12913-020-05673-w (PMC7469361; doi:10.1186/s12913-020-05673-w)
Supplement: Supplementary file 1 — Additional file 1. 28 Articles CoC Literature Review [file 12913_2020_5673_MOESM1_ESM.pdf]

Author	Date	Title	Method	Participants	Setting	Context	Intervention	Outcomes	Continuity of care	Continuity themes	Key themes
Barclay	2014	Improving Aboriginal maternal and infant health services in the 'Top End' of Australia; synthesis of the findings of a health services research program aimed at engaging stakeholders, developing research capacity and embedding change	Mixed-methods health services research program, using a participatory approach	Consumers, midwives, doctors, nurses, Aboriginal Health Workers (AHW), managers, policy makers and support staff	Two large remote Aboriginal communities in the Top End of Australia and the hospital in the regional centre that provided birth and tertiary care	Data were sourced from hospital and health centre records, perinatal datasets and costing datasets, observations of maternal and infant health service delivery and parenting styles, formal and informal interviews and focus groups	A new model of maternity care was introduced by the health service to improve care, informed by the findings of the research. The strategies introduced during the five year program of research were evaluated.	Improvements were made to the acceptability, quality and outcomes of maternity care, which were cost effective. However, a number of system-wide problems remained: unacceptable standards of infant care and parent support, no identifiable relationship between volume and acuity of presentations and staff numbers with the required skills for providing care for infants, and an outpatient model of care. There was a lack of Aboriginal leadership and inadequate coordination between remote and tertiary services.	Yes	Inappopriate resourcing of remote health services, poor coordination, lack of continuity of midwifery carer and discriminatory attitudes and poor practices of clinicians. The intervention described in the paper provided women from two remote communities with continuity of care from a primary midwife.	Remote Aboriginal communities. Top End. New model of maternity care introduced. Aceptability, quality and outcomes of maternity care were improved. However, there was a lack of Aboriginal leadership and inadequate coordination between remote and tertiary services.
Bar-Zeev	2014	Factors affecting the quality of antenatal care provided to remote dwelling Aboriginal women in northern Australia	Mixed method study: 27 semi-structured interviews with clinicians and a retrospective cohort study of Aboriginal women (412) who gave birth between 2004-2006	Clinicians and 412 women	Two remote communities in Northern Territory	The study sought to assess adherence to antenatal care guidelines and examine factors affecting the quality of antenatal care delivery to Aboriginal women in remote settings	None	93% had at least one antenatal visit, which typically took place in the 2nd trimester. Uptake of routine care such as urinalysis and blood pressure monitoring at first visit ranged from 82% to 85%. There was poor adherence to follow-up care for prevalent problems such as anaemia, smoking, UTIs and STIs. The resourcing and organisation of health services and the beliefs, attitudes and practices of clinicians were the major factors affecting quality of care.	Yes	Women in the study were provided with some continuity of carer at the Health Centres during their preganacy with a midwife or district medical officer. Continuity was compromised if women presented to the Health Centres with a non-pregnancy related problem, as a different clinician would treat them. Lack of continuity of care was more frequently reported at the regional hospital, with women seeing multiple different clinicians at each antenatal visit. Discontinuities in communication and documentation that arose from multiple, delayed or absent handovers of patient results or management plans btewen hospital and Health Centres contributed to women receiving inadequate care.	Remore Aboriginal communities. Top End. Majority of women attended antenatal care. Poor adherence to follow-up for identified issues. Resourcing, organisation of health services, and beliefs, attitudes and practices of clinicians were the key elements influencing quality of care.
Bar-Zeev	2012	From hospital to home: the quality and safety of a postnatal discharge system used for remote dwelling Aboriginal mothers and infants in the top end of Australia	Mixed methods study: retrospective cohort study and key informant interviews	413 Aboriginal women and 60 key informants (health, management and administrative staff at health centres and regional hospital maternity, neonatal and paediatric units)	Two remote Aboriginal communities in Northern Territory (maternity unit in a regional public hospital and two remote health centres)	The study aimed to examine the transition of care in the postnatal period from a regional hospital to a remote health service and describe the quality and safety implications for remote dwelling Aboriginal mothers and infants.	None	Poor discharge documentation, communication and coordination between hospital and remote health centres was common. There was a lack of clinical governance and a specific position responsible for postnatal discharge planning in the hospital system, which were identified as serious risks to the safety of the mother and infant. It was noted that the discharge process and service delivery model need to be restructured to reduce the adverse effects on mothers and their infants.	Yes	Participants discussed the importance of improving the links, connections and associations between the two health systems as a means to improving the continuity of care and the communication process. Designated leadership positons including a discharge coordinator were seen as the most effective way of achieving this.	Transition fo care from a regional hospital to a remote health service. Remote setting, Top End. Poor discharge documentation, communication and coordination between hospital and remote health centres. Lack of leadership. Discharge process and service delivery model need to be restructured to reduce adverse outcomes and improve standards of care.
Bertilone	2017	Elements of cultural competence in an Australian Aboriginal maternity program	Surveys and interviews of key informants	Aboriginal Maternity Group Practice Program clients, staff and individuals from partner organisations	South metropolitan Perth	This qualitative study aimed to identify elements of the AMGPP that contributed to the provision of a culturally competent service	None	The partnership model of the AMGPP had a positive impact on the level of culturally appropriate care provided by other health service staff, particularly in hospitals. Two-way learning was highlighted as an important feature. Providing transport, team home visits and employing Aboriginal staff improved access to care. Aboriginal grandmothers successfully brought young pregnant women into the program through their community networks and were able to positively influence health lifestyle behaviours among clients.	Yes	The continuity of care model of the program alowed for ongoing monitoring of clients with risk factors, resulting in better outcomes. The continuity of care model was an important means of engaging clients long term.	Aboriginal Maternity Group Practice Program. Metropolitan Perth. Partnership model and two-way learning with existing maternity services. Positive impact on level of culturally appropriate care. Improved access to care.
Brown	2016	Aboriginal and Torres Strait Islander women's experiences accessing standard hospital care for birth in South Australia - A phenomenological study	Semi-structured interviews (thematically analysed)	14 Aboriginal women who had accessed standard care	Large tertiary maternity teaching hospital in South Australia	Aboriginal women can experience a lack of understanding of their cultural needs when accessing maternity care in the standard hospital care system. The study aimed to explore the lived experiences described by Aboriginal women accessing labour and birth care in the standard hospital care system at a tertiary public hospital.	N/A	6 main themes emerged: knowing what is best and wanting the best for my baby, communicating my way, how they made me feel, all of my physical needs were met, we have resilience and strength despite our hardships, and recognising my culture. Most women identified culturally unsafe practices that they experienced during their care. These could be addressed by the extension of current care plans for include cultural needs, AMIC workers for women from rural and remote areas, AMIC workers on call to assist women and midwives, increased education, employment and retention of Aboriginal midwives, increased review into women's experiences, removal of signs on the door restricting visitors in the birth suite, flexibility in the application of hospital rules and regulations, and changes to birthing services in rural and remote areas so women may not have to relocate for birth.	Yes	Overall, women in the study had mixed feelings about continuity models of care. Some had not been offered a continuity of care model of care, but would have liked the option. Some had been offered the program, but declined because a known midwife was not something they wanted, as they did not wish to have an AMIC worker involved in their care. Privacy and confidentiality were identified as reasons why continuity of care models were not chosen by women.	Aboriginal women in South Australia (mix of rural and remote locations). Culturally unsafe practices were reported by women. Recommendations to extend current care planners to include cultural needs, AMIC workers for women from rural and remote areas, increased education and employment of Aboriginal midwives, flexibility in application of hospital rules and regulations, and expanding rurala nd remote services so that women don't have to relocate for birth.
Brown	2016	Cultural safety and midwifery care for Aboriginal women - A phenomenological study	Semi-structured interviews (thematically analysed)	13 midwives who had provided intrapartum care to birthing Aboriginal women	Large tertiary maternity teaching hospital in South Australia	Culture and culturally appropriate care can contribute to positive health outcomes for Aboriginal women. How midwives provide culturally appropriate care and how the care is experienced by women is poorly understood.	N/A	6 main themes emerged: finding ways to connect with the women, building support networks, managing the perceived barriers to effective care, perceived equity is treating women the same, understanding culture, and assessing cultural needs - urban vs rural/remote Aboriginal cultural needs. Midwives attempt to build support networks around Aboriginal women and the women's support people, but experienced some difficulty differentiating the women's physical needs from their cultural needs. It was proposed that strengthening partnerships between the Aboriginal workforce, the women and the midwives.	Yes	Lack of continuity of care was viewed by midwives as a key barrier to effective care within the mainstream hospital system. Associated barriers included time restraints placed on midwives within a large organisation, the lack of 24/7 support from the Aboriginal workforce and the inflexible policies and procedures of the maternity hospital.	Large tertiary teaching hospital in SA. Semi-structured interviews with midwives. Midwives identifed communication and building support with Aboriginal health workers and families as important. Perceived barriers included misunderstandings around the interpretation of cultural safety in practice.
Corcoran	2017	Models of midwifery care for Indigenous women and babies: A meta-synthesis	Meta-synthesis of qualitative studies	9 qualitative studies (6 from Australia, 3 from Canada)	N/A	There has not been a synthesis of qualitative studies of the model of care to help guide practice development and innovation.	N/A	The major themes identified were valuing continuity of care, managing structural issues, having negative experiences with mainstream services and recognising success. The most positive experiences for women were found with services that provided continuity of care, had strong community links and were controlled by Indigenous communities. However, there were still barriers preventing provision of intrapartum midwifery care in remote communities.	Yes	Continuity of care was one of the major themes that emerged. The most positive experienced for women were with services that provided continuity of care, had strong community links and were controlled by Indigenous communities. Continuity of care enabled relationships between pregnant Indigenous women, midwives and other people involved int heir care, with a fundamental element of building relationshiops being effective communication.	Review of qualitative studies. The major themes identified were valuing continuity of care, managing structural issues, having negative experiences with mainstream services and recognising success. The expansion of midwifery models fo care for Indigenous women and babies could be beneficial in order to improve cultural safety, experiences and outcomes in relation to pregnancy and birth.
Crook	2012	Waminda: mums and bubs program	Narrative description of program	N/A	South Coast Women's Health and Welfare Service Aboriginal Corporation (Waminda), Nowra, NSW	The service is dedicated to providing holistic, culturally competent, safe and secure services for Aboriginal women and children. It has been operating for over 30 years.	Waminda Mums and Bubs program	The program was developed to improve the health outcomes for Aboriginal women and their children. It allows the opportunity to create a trusting and long-term relationship between staff, Aboriginal women and their families. The program offers females lifelong medical and holistic social support. The service responds to community needs, is flexible and has strong partnerships that work towards new and innovative ways of working collaboratively.	Yes	The program described in the paper offers services from pre-conception, antenatal, birthing, postnatal and continuous care by a primary health care team. The holistic approach ensures the program dovetails together with the other services offered by Waminda.	Description of the Mums and Bubs Program. The program focuses in flexibility, having a bi-cultural approach, and partnerships and referral pathways. The service responds to community need and is staffed by skilled Aboriginal and culturally competent non-Aboriginal women.
Gibson-Helm	2018	Identifying evidence-practice gaps and strategies for improvement in Aboriginal and Torres Strait Islander maternal health care	Implementation research study design employing a theory-informed, iterative and interactive dissemination method	Over 100 stakeholders including those from community-controlled organisations, government, general practice health services and research organisations	Australia-wide	The ESP Project drew on aggregated CQI data collected as part of the Audit and Best Practice for Chronic Disease (ABCD) National Research Partnership - a collaboration that brought together PHC services, policy, support organisations and research institutions to guide and support collaborative CQI research to improve the quality of Aboriginal PHC across Australia.	N/A	Four non-communicable disease priority areas were identified: smoking, alcohol, psychosocial wellbeing and nutrition. Strategies to address the priorities included upskilling staff to provide best practice care in priority areas, advocating for availability of healthy food, housing and local referral options, partnering with communities on health promotion projects, systems to facilitate continuity of care and clear referral pathways.	Yes	One of the key strategies identified to improve Aboriginal maternal health care was developing systems to facilitate continuity of care and clear referral pathways.	Stakeholders from a range of professions and organisations participated in the study. Identified barriers and enablers to high-quality care included workforce support, professional development, teamwork, woman-centred care, decision support, equipment and community engagement.
Homer	2012	'It's more than just having a baby' women's experiences of a maternity service for Australian Aboriginal and Torres Strait Islander families	Clinical outcomes collected prospectively and quantitatively analysed. Focus group transcribed and analysed qualitatively.	353 women (clinical records) and 7 women (focus group)	Sydney, NSW	The paper evaluates the Malabar Community Midwifery Link Service from the perspective of Aboriginal women who accessed it.	Malabar Community Midwifery Link Service	353 women gave birth through the service and 40% of babies were identified as Aboriginal or Torres Strait Islander. Over 90% of women had their first visit before 20 weeks of pregnancy. Accessing the service helped women reduce their smoking during pregnancy. Focus group findings showed that women felt the service provided ease of access, continuity of care and caregiver, and trust and trusting relationships.	Yes	The Malabar service is a community-base, primary health care midwifery and child health service. It is a group practise caseload model providing midwifery continuity of care during pregnance, labour and birth and postnatally with referral to child health services after discharge. The importance of continuity of caregiver was highlighted by study participants, with women describing it as "the best part of Malabar". They valued having a person they could call and having caregivers who knew their story.	Malabar Community Midwifery Link Service. Suburbad Sydney. Women felt the service provided ease of access, continuity fo care and caregiver, trust and trusting relationships.
Jongen	2014	Aboriginal and Torres Strait Islander maternal and child health and wellbeing: a systematic search of programs and services in Australian primary health care settings	Systematic literature review	23 publications	N/A	The study reviewed existing knowledge on aboriginal maternal child health programs and services with the objective to advance understanding of the current evidence base and inform MCH service development.	N/A	52% of publications reported on programs and services operating out of Aboriginal Community Controlled Health Organisations. Some intervention studies reported increased antenatal attendance and higher infant birth weights. 13 publications were descriptions of specific programs. Overall, methodological quality varied considerably. Quantitative studies were mostly rated as weak.	Yes	A lack of continuity of care has been identified as a common issue affecting communitication and quality of care in antenatal and postnatal services for Aboriginal women. Fragmented maternity care can increase medical risks and compromise patient safety, causing adverse outcomes for womena nd infants. A focus on continuity of care attempts to address these concerns and ensure quality care that meets the needs of women using these services.	Review of the literature focusing on Maternal Child Health programs. Some studies reported increased antenatal attendance and higher infant birth weighs, but methodological quality was rated as weak.
Josif	2014	'No more strangers': Investigating the experiences of women, midwives and others during the establishment of a new model of maternity care for remote dwelling aboriginal women in northern Australia	Mixed methods: participatory action research (qualitative). Semi-structured interviews, field notes and observations were analysed thematically	66 participants including midwives, Aboriginal Health Workers, department of health staff, and remote dwelling Aboriginal women	Top End of NT	The aim was to describe the experiences of Aboriginal women, midwives and other stakeholders during the establishment of a new model of maternity care for remote dwelling Aboriginal women transferred to a regional centre in NT for maternity care and birth.	Midwifery Group Practice (MGP)	Overall, the new model was viewed as not a perfect system, but one that was changing. Participants agreed that they observed positive changes to the program. The new model was seen as a new way of working and resulted in a very different journey for Aboriginal women using the service. The woman-centred aspect of the new model was appreciated by all stakeholders. Aboriginal women could access continuous care and reported a more positive experience with maternity services.	Yes	The program described resulted in Aboriginal women being able to access continuity of carer in the regional centre for the first time, which resulted in women reporting a more positive experience with maternity services that they received. Women valued being able to get to know a midwife and seeing "the same face".	Remore setting, Top End. Participants reported improvements to the model of maternity care provision. The new model had a woman-centred focus, which was highly valued. Aboriginal women can access continuity of carer for the first time and reported a more positive experience with amternity services.
Josif	2017	The quality of health services provided to remote dwelling aboriginal infants in the top end of northern Australia following health system changes: a qualitative analysis	Semi-structured interviews, participant observation, field notes analysed thematically	25 clinicians providing or managing child health services	Top End of NT	The study describes infant health service quality following health system changes in the area	Health system changes	It was found that many of the issues that were identify prior to the introduction of health system changes persisted, namely: ineffective service delivery, inadequate staffing, and culturally unsafe practices. The six main themes that emerged from the interviews were: very adhoc, swallowed by acute, going under, a flux, a huge barrier, and them and us. Overall, the clinicians viewed the system as very chaotic. Service provision and quality of care were perceived to be inadequate, despite the health system changes. Improvements are needed to the quality, cultural responsiveness and effectiveness of the services.	Yes	Staff turnover and rotation was highlighted by participants as a major contributing factor to poor continuity of care and service quality. Participants described the difficulty in building trust and developing relationships with infants and their families, which was considered key to effective child and family health practice.	Semi-structed interviews with clinicians. Remote Aboriginal communities, Top End. A range of factors impacted quality of care, including ineffective service delivery, inadequate staffing, and culturally unsafe practices. Service provision was perceived as inadequate, despite health system changes,

Kelly	2014	'She knows how we feel': Australian Aboriginal and Torres Strait Islander childbearing women's experience of Continuity of Care with an Australian Aboriginal and Torres Strait Islander midwifery student	Semi-structured interviews	4 Aboriginal women	North Queensland	The study aimed to explore Aboriginal women's experiences of receiving continuity of care from an Aboriginal midwifery student.	Midwifery care provided by Aboriginal midwifery student	Four major themes emerged: communicating our way, the role of relationships, support and assistance, and challenges of the system. Aboriginal women felt that they benefitted as a result of having Aboriginal midwifery students provide continuity of care. The benefits of an Aboriginal midwife providing culturally competent, evidence-based clinical care, as well as having the ability to understand women's social, cultural and practical health care needs were highlighted.	Yes	Participants valued having Aboriginal midwifery students provide continuity of care, with four key themes emerging: communicating our way, the role of relationships, support and assitance and challenges of the system.	Aboriginal women benefitted as a result of having Aboriginal midwifery students provide continuity of care. Health services, in partnership with universities and Aboriginal communities have a vital role to play in the development and expansion of caseload midwifery models with Aboriginal midwives.
Kildea	2016	Remote links: Redesigning maternity care for Aboriginal women from remote communities in Northern Australia - A comparative cohort study	Mixed methods: retrospective cohort study, participatory action research, and prospective cohort study	Cohort 1: 412 Aboriginal women, Cohort 2: 310 Aboriginal women	Top End of NT	The study aimed to assess whether a newly implemented model of care would increase women's access to skilled and culturally appropriate care, continuity of carer and impact positively on quality of care and maternal and infant health outcomes	Midwifery Group Practice (MGP)	The evaluation of the first 21 months of the new MGP service was associated with significant improvements in care outcomes and service uptake. However, despite the improvements the cohorts displayed an excessive burden of illness with very high rates of preterm birth, low birth weight and PPH rates. Despite improved screening rates across a range of areas, over half of women with infections and a third of women with anaemia were not treated in accordance with guidelines. However, the new model of care improved continuity of care, choice, co-ordination of care, collaboration and communication between providers.	Yes	Continuity of midwifery carer was one of the key elements identified and recommended for Birthing on Country models of care. The new model of care opened the pathway for remote-dwelling Aboriginal women to access the regional birth centre, where access relied on care being provided within a continuity of midwifery model.	Remote Aboriginal communities, Top End, and Royal Darwin Hospital. Midwifery Group Practice was associated with significant improvements in many areas reflecting improved access to and quality of care. Targeted program interventions with stronger clinical governance frameworks to improve the quality of care are essential.
Kildea	2018	Birthing on Country (in Our Community): a case study of engaging stakeholders and developing a best-practice Indigenous maternity service in an urban setting	Evaluation of Birthing on Country program	Clients of Birthing in Our Community program	Brisbane, QLD	The Birthing in Our Community program includes 24/7 midwifery care in pregnancy to six weeks postnatal by a midwife supported by Indigenous health workers and a team coordinator, partnership with ACCHS, oversight from a steering committee including Indigenous governance, clinical and cultural supervision, monthly cultural education days, and support for Indigenous student midwives.	Birthing in Our Community program	Three years since the implementation of the program, the program is viewed as successful by clients, and is showing early signs of improved maternal and infant health outcomes. Women participating in the program have higher rates of visits in the first trimester, and lower rates of low birth weight, preterm birth and neonatal nursery admissions.	Yes	The Murri Antenatal Clinic at the Mater Mothers' Hospital was established as a service providing antenatal midwifery and obstetric continuity of care for Indigenous women or women having Indigenous babies, with support from Indigenous Liaison Officers.	Large inner-city hospital and two Aboriginal Community-Controlled Health Services in Queensland. Birthing in Our Community program. The paper showed that Birthing on Country principles can be successfully applied to the urban context. Three years after the establishment of the program, it is proving successful with clients and showing early signs fo improved maternal and infant health outcomes.
Kildea	2010	'Closing the Gap': how maternity services can contribute to reducing poor maternal infant health outcomes for Aboriginal and Torres Strait Islander women	Discussion of role of midwives in improving Aboriginal maternity services in remote areas	N/A	Remote regions of Australia	Health and reproductive outcomes for Aboriginal women worsen with increasing remoteness, where provision of services becomes more challenging. Many women who live remote do not receive care from a skilled provider antenatally, during birth and postnatally.	None	The authors argue for a more intensive, coordinated strategy to improve maternal infant health in rural and remote Australia. Care needs to address social, emotional and cultural health needs, and be as close to home as possible. Midwives can provide comprehensive, quality care within a collaborative team that includes women, community and medical colleagues. Aboriginal women should be encouraged and supported to train as midwives.	Yes	Midwives who have made the change to caseload practice, some of the key principles to sustainability are reported as: the ability to make meaningful relationships with women, offering continuity of carer, the occupational autonomy and flexibility and support at home and work.	Relocating women for birth does not address their cultural needs or self-identified risks and is contributing to poor health outcomes. A more intensive, coordinated strategy to improve maternal infant health in rurala nd remote Australia must be adopted. Care needs to address social, emotional and cultural health needs, and be as close to home as possible.
Kildea	2012	The Murri clinic: a comparative retrospective study of an antenatal clinic developed for Aboriginal and Torres Strait Islander women	Triangulated mixed methods approach: interviews, focus groups, surveys, audit data, routinely collected data	Clinic staff and clients, hospital managers and staff, community stakeholders, representatives from community organisations.	Brisbane, QLD	The study retrospectively compared clinical outcomes of Aboriginal women who attended the Murri Clinic (367) with Aboriginal women attending standard care (414) provided by the same hospital over the same time period.	Murri Clinic: specialist antenatal clinic for Australian Aboriginal women	Women attending the Murri clinic reported high levels of satisfaction, particularly with continuity of care antenatally. However, women reported that this was not sustained during birth and postnatally, leading to feelings of abandonment. Women attending the Murri clinic had higher rates of normal birth compared to women attending usual care.	Yes	Women attending the Murri Clinic reported high levels of satisfaction, specifically with continuity of carer antenatally. However, disappointment with the lack of continuity during labour/birth and postnatally left some women feeling abandoned and uncared for. More work needs to be done to improve coordination and communication between community and tertiary services.	Murri Antenatal Clinic in a tertiary Australian hospital. The majority of women attending the clinic reported high levels of satisfaction, specifically with continuity of carer antenatally. Recommendations for improvement included ongoing cultural competency training for all hospital staff, reducing duplication of services, improving coordination and communication between community and tertiary services, and working in partnership with community-based providers.
Kruske	2006	Cultural safety and maternity care for Aboriginal and Torres Strait Islander Australians	Discussion of provision of maternity services in remote areas in the context of the literature and policies around culture and cultural safety	N/A	Remote regions of Australia	The authors aimed to discuss cultural safety and critique the provision of culturally appropriate maternity services to remote dwelling Aboriginal women in Australia	None	The current provision of services appears largely inadequate. Health system reform at all levels is required in order to deliver culturally safe care, including individual practitioner actions, education of care providers, delivery of services and development of policy.	Yes	To ensure maternity care for Aboriginal women is culturally safe, it is recommended that it should be provided by known practitioners, in a community-based, continuity of carer model. Ideally this should be run through Aboriginal controlled organisations which would replace the often unwelcoming and hostile hospital clinics.	Remote-dwelling Aboriginal women. The current provision of maternity services appears largely inadequate. The provision of culturally safe materntiy care requires health system reform at all levels.
Lack	2016	Narrowing the Gap? Describing women's outcomes in Midwifery Group Practice in remote Australia	Retrospective descriptive study	763 women (306 Aboriginal, 457 non-Aboriginal)	Alice Springs hospital, NT	The papers describes the maternal and newborn outcomes for women accessing midwifery continuity of care in remote Australia	Midwifery Group Practice (MGP)	During the 4 year study period, there were no reported maternal deaths, and lower rates of preterm birth (6%) and low birth weight babies (5%) were found in comparison to population-based data. The findings demonstrate that continuity of midwifery care can be effectively provided to remote dwelling Aboriginal women and appears to improve outcomes for the women and their infants.	Yes	Describes a Midwifery Group Practice that was established in 2009 in a remote town of NT, with the aim of improving outcomes and access to midwifery continuity of care. It was found that continuity of midwifery care can be effectively provided to remote dwelling Aboriginal women and appears to improve outcomes for women and their families.	Remore city in NT. Midwifery Group Practice. The woman's primary midwife was present at 51% of all births and the woman's secondary midwife was presednt at 28% of all births. 16% of births were attended by another MGP midwife who was neither the orimary nor secondary midwife.
Middleton	2017	'Partnerships are crucial': an evaluation of the Aboriginal Family Birthing Program in South Australia	Birth data analysis and interviews	107 health professionals and 20 recent AFBP clients	Nine sites in SA: 3 Adelaide hospitals, 6 regional services in Port Augusta, Whyalla, Port Lincoln, Ceduna, Murray Bridge and Gawler	Evaluation of the implementation and outcomes of the Aboriginal Family Birthing Program (AFBP), which provides culturally competent antenatal, intrapartum and early postnatal care for Aboriginal families in SA	Aboriginal Family Birthing Program	1/3 of SA Aboriginal women attended AFBP services between 2010-12. AFBP women were more likely to be more socially disadvantaged, have poorer pregnancy health, and to have inadequate numbers of antenatal visits compared to Aboriginal women attending other services. However, pregnancy outcomes were similar for the two groups. Interviews with program clients revealed that they highly valued care from an Aboriginal woman. The results suggest that AFBP reaches out to women with the greatest need and provides culturally appropriate and effective care through partnerships.	Yes	The Aboriginal Family Birthing Program has a commitment to continuity of care and includes an AMIC worker in a leadership role and provides education and training for AMIC workers in antenatal, birthing and postnatal care.	Aboriginal families across SA. Around 1/3 of Aboriginal women giving birth in SA atended AFBP services. Clients highly valued care from another Aboriginal woman. Despite challenges, the AFBP reaches out to women with the greatest need, providing culturally appropriate, effective care through partnerships.
Murphy	2012	The Aboriginal Maternal and Infant Health Service: a decade of achievement in the health of women and babies in NSW	Program evaluation	N/A	NSW	The service was established to improve the health of Aboriginal women during pregnancy and decrease perinatal morbidity and mortality among their babies. The service is delivered through a continuity of care model, where midwives and Aboriginal Health Workers collaborate to provide high quality maternity services that are culturally sensitive, women centred, based on primary healthcare principles and provided in partnership with Aboriginal people.	Aboriginal Maternal and Infant Health Service	The evaluation found that the program is achieving its goals with respect to provision of antenatal and postnatal care. It has demonstrated improvements in perinatal morbidity and mortality rates. The implementation of the program resulted in an increased proportion of women attending their first visit before 20 weeks gestation, decreased proportion of preterm births, and improved breastfeeding rates.	Yes	The Aboriginal Maternal and Infant Health Service is delivered through a continuity of care model where midwives and Aboriginal Health Workers collaborate to provide high quality maternity services that are culturally sensitive, woman-centred and provided in partnership with Aboriginal people.	Program established in NSW. The program is achieving its goals and has demonstrated improvements in perinatal morbidity and mortality rates.
Myers	2014	Early childhood nutrition concerns, resources and services for Aboriginal families in Victoria	Focus groups and interviews	35 Aboriginal parents and 45 health and early childhood practitioners	Aboriginal health and early childhood services in urban and regional Victoria	The study sought to investigate child nutrition concerns of Aboriginal families with young children, as well as the training needs of early childhood practitioners.	None	Most frequently identified issues included low rates of breastfeeding, inappropriate introduction of solids, reliance on bottles, sweet drinks and energy-dense foods, poor oral health and overweight. The study identified gaps in service delivery for Aboriginal families with young children, suggesting a need for a coordinated, culturally responsive systems approach to providing support for breastfeeding and child nutrition advice and support for Aboriginal families. Capacity building for staff and supportive systems and policy were also highlighted as important areas to address.	Yes	Practitioners interviewed in the study reported concerns about continuity of care in early childhood services, particularly with access to a Maternal and Child Health nurse or equivalent early childhood health services.	One urban and one rural ACCHO. Concerns about staff training and capacity and access to maternal and child health services were common. The study identified major gaps in service delivery for Aboriginal families with young children and points to the need for a coordinated, culturally responsive systems approach to supporting Aboriginal families.
Parker	2014	'Choice, culture and confidence': key findings from the 2012 having a baby in Queensland Aboriginal and Torres Strait Islander survey	Survey (both quantitative and qualitative data collected)	187 Aboriginal women	Queensland	The aim was to describe the maternity care experiences of Aboriginal women and to identify areas for policy and practice improvements.	None	Women first saw a health professional regarding pregnancy at an average gestation of 7.9 weeks. 67.4% of women attended at least 5 antenatal appointments. 62% indicated that they had experienced at least one stressful life event during pregnancy. 97.3% gave birth in a public hospital and 66% indicated that they did not have a choice as to where they could have their baby. Only half of women with access to an Indigenous specific health service chose to use it. 12.8% of women indicated that they were able to carry out any cultural practices during their pregnancy and birth. Recommendations include enhancement of cultural competency of maternity services, increased access to continuity of midwifery care models, facilitating more choice in care, engaging women in the design and delivery of care.	Yes	Continuity of carer during pregnancy is one of the key themes discussed in the paper. The majority of women indicated that they saw the same health professional during their preganacy. Women commented on their satisfaction with having a known midwife or the same midwife during their pregnancy. Care from a known provider enhanced women's maternity care experiences.	Urban and rural areas across QLD. Women reported high rates of stressful life events in pregnancy, low levels of choice in place of birth and model of care and limited options to carry out cultural practices. Need to enhance cultural competence of maternity services, increase access to continuity of midwifery care models, facilite greater choice in care, work with the strengths of Aboriginal women and communigties and engage women in the design and delivery of care.
Reibel	2016	From consultation to application: Practical solutions for improving maternal and neonatal outcomes for adolescent Aboriginal mothers at a local level	Feasibility study	140 health professionals	WA	The project sought to examine the pregnancy journey for young Aboriginal women and determine the feasibility of an integrated model of care to address known health system issues	Integrated model of care for Aboriginal women	The importance of creating models of antenatal care using a social determinants of health framework was emphasised. Destigmatising young parenthood and providing continuity of caregiver in culturally safe services, with culturally competent health professionals provides a means to encourage engagement with the health system and improve health outcomes for young mothers and their babies.	Yes	Providing continuity of caregiver in culturally safe services with culturally competent professionals provides a means to encourgae engagement with the health system and improves health outcomes for young Aboriginal mothers and their babies. The importance of caregivers developing and maintaining relationships with young Aboriginal mothers is highlighted.	Focus on pregancy among adolescent Aboriginal mothers. The paper discusses the importance of creating models of antenatal care using a social determinants of health framework.

Rumbold	2011	Delivery of maternal health care in Indigenous primary care services: baseline data for an ongoing quality improvement initiative	Cross-sectional baseline audit	535 women	34 Indigenous community health centres in five regions: Top End of NT, Central Australia, Far West NSW, Western Australia and North Queensland	The aim of the study was to describe patterns of delivery of maternity care and service gaps in primary care services in Australian Indigenous communities	None	The proportion of women presenting for their first visit in the first trimester ranged from 34% to 49% between regions, which is why documentation of care early in pregnancy was poor. 46% of known smokers received smoking cessation advice, 52% of women received antenatal education and 51% had investigation for gestational diabetes. There was good documentation of identified problems related to hypertension or diabetes, with over 70% of identified women referred to a GP/Obstetrician. Increasing access to evidence-based screening and health information (particularly around smoking cessation) were identified as opportunities for improvement.	Yes	It was found that in the postnatal period, only 55% of women received breastfeeding advice, and there was poor documentation of advice around smoking, hygiene, injury prevention and SIDS prevention. This indicated that there is a great potential to improve the continuity of care throughout the antenatal and postnatal period. In order to improve continuity in the remote setting, better integration of services or a redesign of care to support community-based services across the spectrum of maternity care would be required.	Five regions (Top End, Central Australia, Far west NSW, WA and North QLD. Documentation of care early in pregnancy was poor. Services had both strengths and weaknesses in the delivery of maternal health care. Increasing access to evidence-based screenign and health information were identified as opportunities for improvement across services.
Rumbold	2008	A review of the impact of antenatal care for Australian Indigenous women and attempts to strengthen these services	Literature review	10 evaluations of antenatal care programs	Australia-wide	The aim was to review evaluations of changes in the delivery of antenatal care for Australian Indigenous women and the impact on care utilisation and quality, birth outcomes and women's views about care.	None	There were wide variations in the design, quality and reported outcomes of each evaluation. There was a lack of consistency in the findings for many outcomes. Modest increases were reported for measures of care utilisations, including accessing services in the first trimester. For birth outcomes, benefits were reported by some but not all care programs for perinatal mortality, preterm birth, birth weight and low birth weight. Women were generally positive about the services, particularly the use of female staff and continuity of care providers.	Yes	Women consistently highlighted the use of female staff and continuity of care as positive aspects of the maternity care that they received.	Literature review of antenatal care programs. Most women reflected positively about their care, includingt he use of female staff and the continuity of care providers. The review highlighted the need for good quality long-term data collection about the health services providing antenatal care for Aborigianl women.
Stamp	2008	Aboriginal maternal and infant care workers: partners in caring for Aboriginal mothers and babies	Semi-structured interviews	5 AMIC workers and 4 midwives	Regional SA	The study investigated the views of AMIC workers and midwives regarding their roles, their partnerships and the Anangu Bibi program, following the first 45 births	Anangu Bibi Family Birthing Program	AMIC workers' role included clinical, cultural and social aspects from the confirmation of pregnancy through to 6-8 weeks after birth. Themes included clinical work, social and emotional support, language and advocacy, clinical benefits and cultural safety. Midwives' role included clinical practice, skill-sharing and mentoring. Midwives were guided by AMIC workers' social, cultural and community knowledge. The development of the partnership essential to the program took commitment and time.	Yes	The Aboriginal Birthing Program had a commitment to continuity of care as one of its key principles. The need for and importance of continuity of care and carer was discussed by care providers, but the difficulties of providing continuity due to staffing and resource issues was acknowledged.	Semi-structured interviews with AMIC workers and midwives. Regional SA. AMIC workers' role included clinical, cultural, social and aspects from the confrimation of pregnancy through to 6-8 weeks after birth. Midwives' role included clinical practice, skill-sharing and mentoring. The paper examines the development of the inter-cultural partnership established to address the health of Aboriginal mothers and babies. Skill sharing and two-way learning engendered mutual respect. Cultural safety was maintained for the Aboriginal mothers and their families as a result of the partnership.
