# Supplementary material for: Aboriginal and Torres Strait Islander family access to continuity of health care services in the first 1000 days of life: a systematic review of the literature
Source: BMC Health Serv Res. 2020 Sep 3;20:829. doi: 10.1186/s12913-020-05673-w (PMC7469361; doi:10.1186/s12913-020-05673-w)
Supplement: Supplementary file 2 — Additional file 2. Prisma Literature Flow Diagram [file 12913_2020_5673_MOESM2_ESM.docx]

**Included**

**Eligibility**

**Identification**

**Screening**

(n=2918)

# of records identified through database searching

(=17)

# of additional records identified through other sources

(n=1147) # of records after duplicates removed

(n=1788) # of records screened

(n=1691) # of records excluded

(n=97) # of full-text articles assessed for eligibility

(n=28) # of studies included in qualitative synthesis

(n=69) # of full-text articles excluded, with reasons:

- Study protocols
- Policy perspectives
- Discussions of risk factors for specific diseases or practices
- Descriptions or evaluations of interventions or programs
- Reports of pregnancy or birth outcome trends/statistics
- Lacking continuity of care across first 1000 days
